# Association of PM _2.5_ Reduction with Improved Kidney Function: A Nationwide Quasiexperiment among Chinese Adults 

**DOI:** 10.34133/2022/9846805

**Published:** 2022-01-15

**Authors:** Yiqun Han, Tao Xue, Frank J. Kelly, Yixuan Zheng, Yao Yao, Jiajianghui Li, Jiwei Li, Chun Fan, Pengfei Li, Tong Zhu

**Affiliations:** ^1^Environmental Research Group, MRC Centre for Environment and Health, Imperial College London, London, UK; ^2^BIC-ESAT and SKL-ESPC, College of Environmental Sciences and Engineering, Center for Environment and Health, Peking University, Beijing 100871, China; ^3^Institute of Reproductive and Child Health/Ministry of Health Key Laboratory of Reproductive Health and Department of Epidemiology and Biostatistics, School of Public Health, Peking University, Beijing, China; ^4^Center of Air Quality Simulation and System Analysis, Chinese Academy of Environmental Planning, 100012 Beijing, China; ^5^China Center for Health Development Studies, Peking University, Beijing, China; ^6^College of Computer Science and Technology, Zhejiang University, Hangzhou, China; ^7^Computer Center, Peking University and Peng Cheng Laboratory, China; ^8^Advanced Institute of Information Technology, Peking University, China

## Abstract

*Background*. Increasing evidence from human studies has revealed the adverse impact of ambient fine particles (PM _2.5_) on health outcomes related to metabolic disorders and distant organs. Whether exposure to ambient PM _2.5_ leads to kidney impairment remains unclear. The rapid air quality improvement driven by the clean air actions in China since 2013 provides an opportunity for a quasiexperiment to investigate the beneficial effect of PM _2.5_ reduction on kidney function.*Methods*. Based on two repeated nationwide surveys of the same population of 5115 adults in 2011 and 2015, we conducted a difference-in-difference study. Variations in long-term exposure to ambient PM _2.5_ were associated with changes in kidney function biomarkers, including estimated glomerular filtration rate by serum creatinine (GFR _scr_) or cystatin C (GFR _cys_), blood urea nitrogen (BUN), and uric acid (UA).*Results*. For a 10  *μ*g/m ^3^ reduction in PM _2.5_, a significant improvement was observed for multiple kidney functional biomarkers, including GFR _scr_, BUN and UA, with a change of 0.42 (95% confidence interval [CI]: 0.06, 0.78) mL/min/1.73m ^2^, -0.38 (-0.64, -0.12) mg/dL, and -0.06 (-0.12, -0.00) mg/dL, respectively. A lower socioeconomic status, indicated by rural residence or low educational level, enhanced the adverse effect of PM _2.5_ on kidney function.*Conclusions*. These results support a significant nephrotoxicity of PM _2.5_ based on multiple serum biomarkers and indicate a beneficial effect of improved air quality on kidney function.

## 1. Introduction

Chronic kidney disease (CKD) has a significant and increasing impact on the world’s population (*1*). In 2017, the recorded cases of CKD were 697.5 million with an average prevalence of 9.1% globally, resulting in 35.8 million disability-adjusted life-years (DALYs) (*2*). The symptoms of CKD are usually latent initially, before becoming more apparent manifesting as cardiovascular disease (CVD), edema, bone disease, anemia, and nerve damage. Finally, this leads to kidney failure, known as end-stage kidney disease (ESRD) when regular dialysis treatment or a kidney transplant is the only options for survival (*1*). Declining kidney function is a key characteristic and diagnostic indicator which identifies the onset and development of CKD. Traditional risk factors of CKD and kidney dysfunction, such as diabetes and hypertension (*1*), cannot fully explain the geographic heterogeneity of the disease, and it is suggested that other drivers including air pollution may account for the variation (*3*, *4*).

The air pollutant causing most health concerns is the particulate matter with aerodynamic diameter smaller than 2.5  *μ*m (PM _2.5_) which has been well documented for its adverse cardiorespiratory and metabolic effects (*5*). It has been proposed that the underlying biological mechanisms, such as systemic inflammation, oxidative stress, and vascular endothelial dysfunction, may potentially damage distant organs including the kidneys, which is a highly vascularized organ of the human circulatory system (*6*).

Increasing evidence from human studies has revealed associations between air pollution and declining kidney function indicators such as the prevalence (*7**–**9*), incident and progression of CKD (*8*, *10*), and decreased estimated glomerular filtration rates (eGFR) (*7*, *8*, *11**–**13*); although, the findings remain inconsistent. A study which included 1.1 million adults from the US Medicare program reported that the annual concentration of county-level PM _2.5_ was positively associated with the prevalence of diagnosed CKD (*9*). However, this conclusion has not been repeated in three other large cross-sectional studies (*7*, *8*, *14*). On the other hand, the evidence from cohort studies is consistent that long-term exposure to PM _2.5_ is significantly associated with the incident and progression of CKD (*8*) and the risk of eGFR decline (*10*, *12*). To date, no evidence has been provided to examine if PM _2.5_ reduction could beneficially impact kidney function in human.

The Clean Air Action Plan, initiated in 2013 by the Chinese government, was a bold nationwide policy aiming at tackling the severe air pollution problem in China that was leading to about 1 million premature deaths every year (*15*). The plan consists of ten key measures including some important policies as reducing emissions from industrial facilities and vehicles and improving the efficiency of fuel usage (*16*). As a consequence, annual PM _2.5_ concentrations in mainland China have decreased significantly by 32%, from 67.4  *μ*g/m ^3^ in 2013 to 45.5  *μ*g/m ^3^ in 2017 (*15*). This rapid change provides an opportunity of designing quasiexperiments to investigate the beneficial effect of air pollution reduction on the human health in the Chinese population (*17**–**19*). Based on repeated nation-scale surveys launched before and after the Clean Air Action Plan in 2011 and 2015 on the same population of 5115 adults, we conducted a difference-in-difference study to investigate the causal relationship between chronic exposure to ambient PM _2.5_ and changes in kidney function.

## 2. Methods

### 2.1. Population Data

The population in this study was based on the project “The China Health and Retirement Longitudinal Study (CHARLS),” which provided a public database (http://opendata.pku.edu.cn/) with a wide range of information from socioeconomic status to health conditions in Chinese people aged 45 and over (*20*). To ensure sample representativeness, ~20,000 participants were recruited based on a complex four-stage sampling approach across the country. A detailed introduction and protocol of the project is provided in the CHARLS document (*21*). Briefly, three waves of repeated surveys were launched in the years 2011, 2013, and 2015, and most of the surveys were conducted during summer season (Figure S1). The analysis of this study relied on the data collected from waves 1 and 3, because blood samples were not collected in wave 2. Demographic background and social economic information of all participants were collected using follow-up questionnaire qualified enumerators. Blood samples were also collected by well-trained staff. 

### 2.2. Kidney Function Measurement

The evaluation of kidney function was based on four clinical biomarkers measured in fast blood samples, namely, serum creatinine (SCR), cystatin C (CYS), blood urea nitrogen (BUN), and uric acid (UA). Samples were sent to a central national laboratory and stored -80°C until assay. Strict standards were applied from sample transportation, storage, to the measurement and quality control, with details in supplement (S1.1). 

Decline in glomerular filtration rate (GFR) was deemed as the golden standard for the clinical diagnosis of CKD (*1*). In this study, we applied the two equations to calculate eGFR by SCR (GFR _scr_) or CYS (GFR _cys_), based on age and sex (details in S1.2) (*22*). In a comparative study (*21*) for Chinese adults, the utilized equations were proofed to outperform other eGFR approaches suggested by the Chronic Kidney Disease Epidemiology Collaboration (*23*). BUN and UA can also be utilized as biomarkers of kidney impairment, because these two biomarkers reflect the concentrations of waste products generated from protein and purine metabolism, respectively. The elevated concentrations of BUN and UA are usually seen in patients with reduced GFR (*24*, *25*). Since BUN and UA may also be indicative for other diseases (e.g., gouty arthritis), in this study, they act as secondary biomarkers for kidney function. Major conclusions from our analyses should be drawn from the eGFR results. 

### 2.3. Exposure Data

Similar to our previous studies, we evaluated the long-term exposure to PM _2.5_ and temperature for CHARLS subjects according to the reanalyzed environmental database (*26*, *27*). Details on exposure assessment are documented in supplement (S1.3). 

### 2.4. Study Design and Statistical Analyses

This study applied a difference-in-difference method to examine the causal effect of PM _2.5_ on kidney function. The same approach has been applied in our previous analyses (*17*, *19*) and briefly introduced in supplement (S1.4). For this study, the difference-in-difference analysis was parameterized using the following equation:

(1)ΔBiomarkeri=ΔPM2.5,iβ+Δxiγ1+xiγ2+εi,

where i denotes the individual index; *Δ*PM _2.5,*i*_ denotes the temporal change in the exposure level from pre- to postclean air actions for the i*-*th adults; *Δ*Biomarker *_i_* denotes the corresponding temporal change in a targeted biomarker; *Δx _i_* denotes the temporal changes in the longitudinal covariates (i.e., inconstant variables, including marriage, smoking, drinking, indoor temperature maintenance, cooking energy type, and body weight, as shown in Table 1); *x _i_* denotes the baseline covariates (i.e., constant variables, including residence, sex, and education); and *β* and ***γ***_1_ and ***γ***_2_ denotes the corresponding regression coefficients. For categorical longitudinal variables, *Δ****x****_i_* denotes a new categorical variable, coded by combination of measurements in the two waves (for example, between the two surveys, a person who quit smoking was coded as yes-no, and a person who started to smoke was coded as no-yes); for continuous longitudinal variables, *Δx _i_* denotes the difference between the two measurements. Given the possibility that the studied subjects were not completely randomly distributed along different levels of *Δ*PM _2.5_, we applied the inversed probability weights, which were derived using the R package *ipw*. The probability of *Δ*PM _2.5_ was estimated using a regression model that incorporated covariates of residence, sex, education, age, BMI, temperature variation, body weight change, and an indicator of regional developmental level and gross domestic product per capita. The effect of PM _2.5_ on kidney function was evaluated as (10×β), i.e., the change in biomarkers for a 10  *μ*g/m ^3^ increment of PM _2.5_ in the long-term exposure.*Δx _i_ γ *_1_ aims to control for the potential confounding effects from the longitudinal changes in the study population, such as the lifestyle (drinking and smoking) changes.*x _i_ γ *_2_ aims to control for the heterogenous temporal trend in the kidney function, which is assumed to progress in temporal patterns different by demographic characteristics (e.g., educational level). Since *Δ*Biomarker *_i_* for each of the four biomarkers was distributed in bell shape (Figure S2), there was no clear evidence to model the outcomes in transformed scale. 

**Table 1:** Statistics of the population characteristics.

Constant variables	Categorical variable	Group	Person (n, prevalence %)		
	Total		5115 (100%)		
	Residence	Rural	3387 (66.2%)		
		Urban	1728 (33.8%)		
	Region	Midwest	1771 (34.6%)		
		North	1439 (28.1%)		
		Southeast	1905 (37.2%)		
	Sex	Female	2830 (55.3%)		
		Male	2284 (44.7%)		
		Unknown	1 (0.02%)		
	Education	Elementary and below	3619 (70.8%)		
		High and above	477 (9.33%)		
		Middle	1017 (19.9%)		
		Unknown	2 (0.04%)		
	Continuous variable		Mean (SD, IQR, person n)		
	Average body mass index (BMI, kg/m ^2^)		23.9 (3.8, 21.4 ~ 26.1, 5115)		
	Age at 2011 (year)		58.8 (8.9, 52.0 ~ 65.0, 5114)		

Inconstant variables	Categorical variable	Group	Person-visit (N, prevalence %)		
			Total	Wave before intervention	Wave after intervention
	Marriage	No	1709 (16.7%)	763 (14.9%)	946 (18.5%)
		Yes	8520 (83.3%)	4352 (85.0%)	4168 (81.5%)
		Unknown	1 (0.01%)	0 (0%)	1 (0.02%)
	Smoking	No	7246 (70.8%)	3645 (71.3%)	3601 (70.4%)
		Yes	2981 (29.1%)	1467 (28.7%)	1514 (29.6%)
		Unknown	3 (0.03%)	3 (0.06%)	0 (0%)
	Drinking	Frequent	2505 (24.5%)	1244 (24.3%)	1261 (24.7%)
		Never	6886 (67.3%)	3455 (67.6%)	3431 (67.1%)
		Rare	831 (8.1%)	413 (8.1%)	418 (8.2%)
		Unknown	8 (0.1%)	3 (0.06%)	5 (0.10%)
	Indoor temperature maintenance	Very hot	111 (1.1%)	88 (1.7%)	23 (0.5%)
		Hot	970 (9.5%)	561 (11.0%)	409 (8.0%)
		Bearable	8688 (84.9%)	4308 (84.2%)	4380 (85.6%)
		Cold	277 (2.7%)	122 (2.4%)	155 (3.0%)
		Very cold	18 (0.2%)	16 (0.3%)	2 (0.04%)
		Unknown	166 (1.6%)	20 (0.4%)	146 (2.9%)
	Cooking energy type	Clean	4701 (46.0%)	2043 (39.9%)	2658 (52.0%)
		Unclean	5440 (53.2%)	3040 (59.4%)	2400 (46.9%)
		Unknown	89 (0.9%)	32 (0.6%)	57 (1.1%)
	Continuous variable		Mean (SD, median, IQR)		
	GFR _scr_ (mL/min/1.73 m ^2^)		87.7 (10.1, 89.5, 83.0 ~ 94.6)	89.1 (9.7, 90.8, 84.3 ~ 95.8)	86.3 (10.3, 88.2, 81.8 ~ 93.2)
	GFR _cys_ (mL/min/1.73 m ^2^)		87.8 (19.9, 85.2, 75.7 ~ 98.7)	82.7 (19.0, 81.0, 71.1 ~ 92.0)	93.0 (19.5, 92.0, 80.2 ~ 105.1)
	Serum creatinine (SCR, mg/dL)		0.8 (0.2, 0.7, 0.6 ~ 0.9)	0.8 (0.2, 0.7, 0.6 ~ 0.9)	0.8 (0.2, 0.8, 0.7 ~ 0.9)
	Cystatin C (CYS, mg/L)		0.9 (0.3, 0.9, 0.8 ~ 1.0)	1.0 (0.3, 1.0, 0.9 ~ 1.1)	0.9 (0.2, 0.9, 0.7 ~ 1.0)
	Blood urea nitrogen (BUN, mg/dL)		15.6 (4.5, 15.0, 12.5 ~ 18.2)	15.6 (4.4, 15.0, 12.5 ~ 18.0)	15.7 (4.6, 14.8, 12.3 ~ 18.2)
	Uric acid (UA, mg/dL)		4.6 (1.3, 4.5, 3.7 ~ 5.4)	4.4 (1.2, 4.2, 3.5 ~ 5.1)	4.9 (1.4, 4.8, 3.9 ~ 5.7)
	Body weight (kg)		59.4 (11.7, 58.4, 51.5 ~ 66.3)	59.3 (11.4, 58.3, 51.4 ~ 66.2)	59.5 (11.9, 58.5, 51.5 ~ 66.5)
	PM _2.5_ (*μ*g/m ^3^)		58.9 (18.5, 59.1, 44.1 ~ 73.9)	63.1 (18.9, 64.8, 49.2 ~ 78.8)	54.7 (17.0, 55.9, 40.7 ~ 69.7)

In sensitivity analyses, we first examined whether the estimated effect varied with model settings: (1) different choices of adjusted covariates and (2) incorporation of the inversed probability weights or not. Second, we explored how the estimated effect was heterogeneous between different subpopulations using interaction analyses. Third, linearity of the effect was tested with details in supplement (S1.5). Fourth, we explored how the estimated effect of PM _2.5_ was varied between subjects with different baselines of kidney function. The baseline-varying model, which is similar to the nonlinear model, has been applied in our previous study (*28*) and is documented in the supplement (S1.5). Fifth, we explored whether the estimated associations were sensitive to the potential heterogeneity between different survey waves or the skewed distributions of the biomarker levels. To test that, we first normalized the measurements (i.e., $\text{normalized biomarke}{\text{r}}_{i}=\left(\text{biomarke}{\text{r}}_{i}-\text{mean}\right)/\text{SD}$) within each wave and then utilized their differences as new dependent variables to reexamine the effect of PM _2.5_. Sixth, we conducted two sensitivity analyses to evaluate the exposure measurement errors caused by using city-level average to assess the PM _2.5_ exposure. We applied a well-developed bootstrap method (Supplement S1.6), which has been applied in our previous studies, to quantify the exposure measurement error (*17*, *29*). We also further lowered the error by deriving city-level PM _2.5_ concentrations specifically for urban or rural area (Supplement S1.7) and reestimated the associations. Finally, we also conducted post hoc analyses to examine whether the associations were sensitive to the choice of time window for exposure assessment. We repeated our main models using alternative city-level PM _2.5_ concentrations averaged within 1, 2, 3, or 4 years preceding the survey time. All the statistical analyses were performed using R (version 3.4.1), and the significance level was set as p<0.05. 

## 3. Results

### 3.1. Descriptive Statistics

In total, this study involved 5115 eligible adults, who have completed the survey waves 1 and 3 (i.e., pre- and postclean air actions, respectively (Figure S1). Among them, there were 4911, 3841, 5109, or 5113 pairs of valid records on SCR, CYS, BUN, and UA, respectively. Mean age of the studied population at 2011 was 58.8 (standard deviation=8.9) years old. In the survey wave 1, the mean values of GFR _scr_, GFR _cys_, BUN, or UA were 89.1 (9.7) mL/min/1.73 m ^2^, 82.7 (19.0) mL/min/1.73m ^2^, 15.6 (4.4) mg/dL, or 4.4 (1.2) mg/dL, respectively. In the wave 3, those values were 86.3 (10.3) mL/min/1.73 m ^2^, 93.0 (19.5) mL/min/1.73m ^2^, 15.7 (4.6) mg/dL, or 4.9 (1.4) mg/dL, respectively. Between the two waves, mean body weight slightly increased from 59.3 (11.4) kg to 59.5 (11.9) kg, and the mean exposure concentration of PM _2.5_ decreased from 63.1 (18.9) *μ*g/m ^3^ to 54.7 (17.0) *μ*g/m ^3^. Across the studied cities, we found a significantly decreasing trend of 4.95 (95% confidence intervals [CI]: 0.79~9.11) *μ*g/m ^3^/year from 2013 to 2016 in the monthly concentrations of ambient PM _2.5_ after the intervention (Figure S1). In contrast, before the intervention, there was no significant trend in PM _2.5_ (0.29  *μ*g/m ^3^/year; 95% CI: -3.66~4.25  *μ*g/m ^3^/year) from 2009 to 2012. Spatial distributions of the PM _2.5_ trends are documented in our previous publication (*17*). Detailed summaries of the studied population are presented in Table 1. 

### 3.2. Associations

To illustrate the design of a difference-in-difference study, we first conducted a preliminary analysis (Figure S2). The actual changes in PM _2.5_ exposure (*Δ*PM _2.5_ equals the concentration of PM _2.5_ in 2015 minus the concentration in 2013) varied in different cities. Subjects were divided into two groups, namely, those who lived in the areas with a *Δ*PM _2.5_ below its upper quartile ($\Delta \text{P}{\text{M}}_{2.5}<-5.12\mu \text{g}/{\text{m}}^{3}$) as the treatment group, referring to a more efficient effect of the clean air actions and the rest as the control (Figure S2, upper panel). Because different magnitudes of PM _2.5_ reduction were mostly driven by emission-control policies, the between-group difference in the temporal change in a biomarker (*Δ*Biomarker) could be utilized to reveal the policy’s effect. The lower panel of Figure S2 suggests the intervention was associated to a positive change in GFR _scr_ (0.22 mL/min/1.73m ^2^) and GFR _cys_ (0.88 mL/min/1.73m ^2^) but a negative change in BUN (-0.14 mg/dL) or UA (-0.16 mg/dL). Because the reduction in GFR _scr_ or GFR _cys_, and the increment of BUN or UA suggests a kidney impairment, the results consistently revealed the benefit of PM _2.5_ reduction on kidney function. 

Considering the PM _2.5_ reduction as continuous, the between-group comparison of *Δ*Biomarker could be converted into a regression analysis (i.e., the difference-in-difference model), which enable a quantitative examination on the association between *Δ*Biomarker and *Δ*PM _2.5_, as shown in Figure S3. According to the fully adjusted models (Table 2), an increment of 10  *μ*g/m ^3^ in PM _2.5_ was associated to a change of -0.42 (95% CI: -0.78, -0.06) mL/min/1.73m ^2^, 0.02 (-1.16, 1.20) mL/min/1.73m ^2^, 0.38 (0.12, 0.64) mg/dL, and 0.06 (0.00, 0.12) mg/dL for GFR _scr_, GFR _cys_, BUN, and UA, respectively. The estimated effects of PM _2.5_ were not sensitive to different model settings. The findings based on GFR _scr_, BUN, and UA consistently suggested a significantly adverse effect of PM _2.5_ exposure on kidney function. The large uncertainties in the estimated effect on GFR _cys_ might be due to its relatively small sample size (Table 1). 

**Table 2:** The association between PM _2.5_ and kidney function biomarkers, estimated directly from the fully adjusted regression or after a further correction using the bootstrap method.

		Biomarker change ^*^ for 10 *μ*g/m ^3^ increment in PM _2.5_ with 95% percentile intervals	
Method		Regression estimation (no correction for exposure measurement error)	Bootstrap estimation (after correction for exposure measurement error)
Biomarker change	GFR _scr_ (mL/min/1.73m ^2^)	**-0.421 (-0.784, -0.058)** ^**#**^	**-0.396 (-0.766, -0.014)**
	GFR _cys_ (mL/min/1.73m ^2^)	0.018 (-1.161, 1.197)	-0.002 (-1.232, 1.149)
	BUN (mg/dL)	**0.379 (0.118, 0.640)**	**0.358 (0.098, 0.620)**
	UA (mg/dL)	**0.060 (0.003, 0.116)**	**0.057 (0.002, 0.115)**

#The estimated associations, which are statistically significant (p<0.05), are highlighted by bolded numbers. ^*^The positive/negative change means increase/decrease in a biomarker. For GFR _scr_ or GFR _cys_, a negative association indicates for the toxic effect of PM _2.5_; For BUN or UA, a positive association indicates for the toxic effect of PM _2.5_.

### 3.3. Sensitivity Analyses

**Figure 1:**
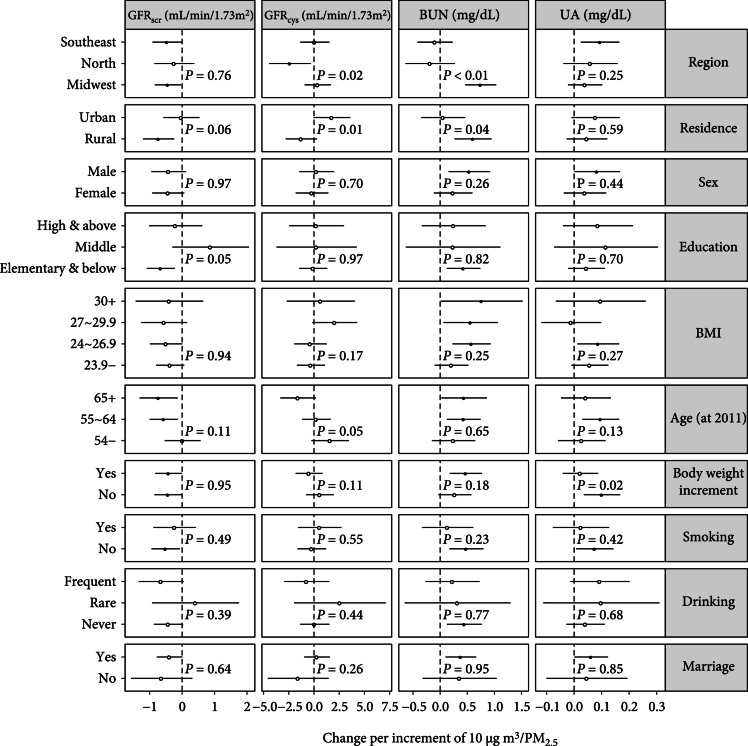
The subpopulation-specific effects of PM _2.5_ on the biomarkers of kidney function. The fully adjusted model incorporated both the baseline covariates and the longitudinal covariates. The dots denote significant associations, and the circles denote nonsignificant ones. Longitudinal covariates denoted temporal changes in the inconstant variables (i.e., body weight, marriage, drinking, smoking, cooking energy type, and indoor temperature maintenance); the baseline covariates denoted values of the longitudinal variables in the baseline wave and the constant variables (i.e., residence, sex, education, age at 2011, and average BMI). Along x-axis, the positive/negative change means increase/decrease in a biomarker. For GFR _scr_ or GFR _cys_, a negative association indicates for the toxic effect of PM _2.5_; for BUN or UA, a positive association indicates for the toxic effect of PM _2.5_.

Figure 1 presents the results from subgroup analyses. We found that the estimated effects were not significantly varied within the most of subpopulation indicators, except for the variables related to the socioeconomic status and age. Compared to the urban residents, rural people were more susceptible to the effect of PM _2.5_ on GFR _scr_ (p=0.06), GFR _cys_ (p=0.01), and BUN (p=0.04). The association between PM _2.5_ and GFR _scr_ was also varied with education level at marginal significance (p=0.05), with enhanced susceptibility in the lowest educated subpopulation. We also observed a trend that aging could enhance the associations between PM _2.5_ and GFR _scr_ (p=0.11) and GFR _cys_ (p=0.05). Additionally, directions of the estimated associations reported by the nonlinear models were in consistent with the results from the main (linear) models (Figure S4). Furthermore, the baseline-varying effect models suggested that the adults with normal kidney function (e.g., $\text{GF}{\text{R}}_{\text{scr}}>\sim 80\text{mL}/\min/1.73{\text{m}}^{2}$ or BUN<~20mg/dL), who might be exposed to less competing risk factors (e.g., alcohol usage), could be more susceptible to the toxic effect of PM _2.5_, compared to those with poor kidney function (Figure S5). Finally, after normalizing the biomarkers, we still observed significant effect of PM _2.5_ on GFR _scr_, BUN, and UA (Figure S6). 

We utilized a bootstrap method to evaluate how the exposure errors influenced the association estimates. The bootstrapped results were presented in Table 2. Generally speaking, the bootstrapped results were statistically comparable with the estimates before correcting the measurement errors. We also reevaluated the associations by urban-or-rural-specific exposures, and the reestimated results were statistically comparable with those from the main models for all biomarkers, except for UA (Figures S3-4). After incorporating the type of residential community into the exposure assessment, the association between PM _2.5_ and UA was no longer statistically significant (Figure S3b). Given that, the effect estimations from our major outcomes, i.e., GFR _scr_ and BUN, were not significantly changed by the limitation of using city-level PM _2.5_ data. Additionally, the bootstrapped method tends to report weaker associations between PM _2.5_ and kidney function biomarkers, which suggests that the measurement errors in exposure may lead to underestimated uncertainties embedded in the associations. Finally, we also found for exposure in a longer term, the effects for per-unit change in PM _2.5_ tend to show larger point estimates but with wider uncertainty ranges (Figure S7). Using a longer time-window for exposure might be more representative for the chronic effect of PM _2.5_ on kidney function, but less representative to show the improved air quality. 

## 4. Discussion

### 4.1. Summary of Previous Findings

Current evidence regarding the adverse effect of PM _2.5_ on kidney remains scares and inconsistent. A recent meta-analysis reviewed literatures on this topic from archive inception to October 2019 indicated that, per 10  *μ*g/m ^3^ increase in the long-term exposure to PM _2.5_, the pooled effect on the eGFR decline was insignificant, estimated to be −4.11 (95% CI: –12.64, 4.42) (*30*). However, this review acknowledged that the analysis was based on only two available studies which may lead to large uncertainties. Specifically, a cohort study based on 669 US male veterans observed that a 2.1  *μ*g/m ^3^ IQR higher 1-year PM _2.5_ was significantly associated with decreased change in eGFR of -1.87 (–2.99, –0.76) mL/min/1.73 m ^2^, (*12*) while another cross-sectional study based on 21,656 Taiwanese concluded an insignificant association (*7*). To be noted, three other studies have also investigated the similar hypothesis but were not included in the meta-analysis (*8*, *10*, *11*). Two cross-sectional studies in African Americans and Taipei city residents reported insignificant associations (*8*, *11*), while another nationwide cohort study among 2,482,737 US veterans found that a 10  *μ*g/m ^3^ increase in PM _2.5_ concentration was associated with significant increased risk of $\text{eGFR}<60\text{mL}/\min/1.73{\text{m}}^{2}$ (hazard ratio (HR) equals 1.21 (1.14 to 1.29)) and eGFR decline≥30% (HR equals 1.28 (1.18, 1.39)) (*10*). The result from our study further confirmed the potential kidney toxicity of PM _2.5_; although, the estimated effect [-0.42 (-0.78, -0.06) mL/min/1.73m ^2^ change in GFR _src_ per an increment of 10  *μ*g/m ^3^ in PM _2.5_] was smaller compared to the findings from previous study (*12*). 

Apart from the decline in eGFR, evidence using other outcomes was also supportive for an adverse effect of PM _2.5_ on kidney function. Significant associations between chronic exposure to PM _2.5_ with the prevalence and incident of CKD and progression of ESRD were suggested from several nationwide cross-sectional and cohort studies (*7**–**10*), in which the definition of CKD and ESRD was largely based on the value of eGFR by SCR. Additionally, a unique study based on an 11-year collection of 71,151 native kidney biopsies from 938 hospitals across 282 cities in China observed that long-term exposure to PM _2.5_ was associated with an increased risk of membranous nephropathy, the second leading type of glomerulopathy that contributed to 23.4% of all cases (*31*). Besides, the observational studies, a few biological evidences also indicate an association between PM _2.5_ exposure and impaired kidney function (Supplmental S1.8). 

### 4.2. Implications

Long-term exposure to ambient PM _2.5_ has been known to cause premature deaths by increasing the risks of cardiorespiratory diseases. GBD study in 2016 estimated the global deaths and DALY attributed to PM _2.5_ was about 4.1 million and 105.7 million, respectively (*32*). However, these numbers could underestimate the health impacts from air pollutants, because a recent study on the association between all-cause mortality and PM _2.5_ showed that cardiorespiratory effects might not be the only explanation behind the mortality burden attributable to PM _2.5_ (*33*). One recent study estimated that the annual global toll of CKD attributable to ambient PM _2.5_ exposure is significant with 6.9 million incident cases of CKD and 11.4 million DALYs (*3*). representing about 10% of burden of disease reported from GBD 2016. In accordance with other evidence (*7**–**10*), our findings further reveal a causal linkage between PM _2.5_ exposure and kidney impairment and suggest that kidney disease could be a nonnegligible outcome of the poor air quality. Future evaluations should incorporate the effects of PM _2.5_ on kidney function and diseases, to accurately quantify the risks from nonoptimal air quality. 

### 4.3. Limits

First, this study is based on all-available observations from a series of preestablished surveys with many general aims, which were not focused on kidney function specifically. Its representativeness depends on the missing patterns in the dataset. However, from the publicly available version of CHARLS, information on the missingness is limited, which can introduce potential bias into (e.g., survival bias), or lower the representativeness of our findings. Second, we reasoned the estimated effect might be biased due to exposure misclassification by the usage of monthly and city-level averages of PM _2.5_ concentrations; although, this is the best dataset available for analysis because the CHARLS did not release the population data with a higher spatiotemporal resolution, in order to protect personal privacy. Finally, although the difference-in-difference analysis controls for some unchanged risk factors on kidney function (e.g., generic defects) by the study design itself, our findings could be undermined by the unmeasured longitudinal confounders. Such confounders can be environmental factors (i.e., lead) that shared the common emission sources of the ambient PM _2.5_. The study design also controlled for the longitudinal factors that were progressed with time in the same pattern. Age is an example of such factor, if we assume it affects kidney function linearly. However, the design cannot fully control for nonlinear risk factors. For instance, the effect of 4-year’s aging could be varied between individuals of different generations. Compared to conventional cross-sectional analyses, our difference-in-difference design is more capable to reveal a causal effect of PM _2.5_, but the causality of our findings is not conclusive and should be reexamined in future. 

## 5. Conclusion

Based on a quasiexperiment design, this study provides a strong evidence for the linkage between chronic exposure to ambient PM _2.5_ and kidney impairment. The findings suggest clean air actions applied in China brings rapid improvement in air pollution and can lead to beneficial health effect by reducing the impact of kidney diseases.

## Data Availability

All the analyses in this study are based on publicly-available datasets. Health outcomes can be freely obtained from http://opendata.pku.edu.cn/; and exposure data can be freely obtained from http://tapdata.org.cn/.

## Supplementary Materials

Supplementary Materials Supplemental Methods Table S1: the assay methods for the kidney function measurements. Table S2: equations for GFR estimation. Figure S1: two waves of health surveys before and after intervention (black bars) with the time series of monthly ambient PM _2.5_ averages (red lines) across the study period. Figure S2: illustration of the study design: a preliminary difference-in-difference analysis by treatment and control groups. Figure S3: estimated effects of PM _2.5_ on the biomarkers of kidney function by different model runs. Figure S4: estimated nonlinear effects of PM _2.5_ on the biomarkers of kidney function. Figure S5: estimated baseline-varying effects of PM _2.5_ on the biomarkers of kidney function. Figure S6: estimated effects of PM _2.5_ on the normalized biomarkers of kidney function by different model runs. Figure S7: estimated effects of PM _2.5_ on the normalized biomarkers of kidney function by different time windows (i.e., periods of 1-4 years before biomarker measurement) for exposure.
