# Supplementary material for: Association of PM 2.5 Reduction with Improved Kidney Function: A Nationwide Quasiexperiment among Chinese Adults
Source: Health Data Sci. 2022 Jan 15;2022:9846805. doi: 10.34133/2022/9846805 (PMC10904065; doi:10.34133/2022/9846805)
Supplement: Supplementary Materials — Supplemental Methods Table S1: the assay methods for the kidney function measurements. Table S2: equations for GFR estimation. Figure S1: two waves of health surveys before and after intervention (black bars) with the time series of monthly ambient PM 2.5 averages (red lines) across the study period. Figure S2: illustration of the study design: a preliminary difference-in-difference analysis by treatment and control groups. Figure S3: estimated effects of PM 2.5 on the biomarkers of kidney function by different model runs. Figure S4: estimated nonlinear effects of PM 2.5 on the biomarkers of kidney function. Figure S5: estimated baseline-varying effects of PM 2.5 on the biomarkers of kidney function. Figure S6: estimated effects of PM 2.5 on the normalized biomarkers of kidney function by different model runs. Figure S7: estimated effects of PM 2.5 on the normalized biomarkers of kidney function by different time windows (i.e., periods of 1-4 years before biomarker measurement) for exposure. [file 9846805.f1.docx]

**Supporting Information**

**Associate PM_2.5_ reduction with kidney function improvement: a nationwide quasi-experiment among Chinese adults**

**Table of Contents**

- **Supplemental Methods**
- **Supplemental Tables**
- **Supplemental Figures**
- **References**

**S1 Supplemental Methods**

**S1.1 Kidney function measurements**

Three tubes of venous blood were collected from each participant by medically-trained staﬀ from the China CDC, based on a standard medical protocol. For most participants, the blood collection was done at centralized locations, which is either in district CDC in urban areas, or in county CDC/town village health centres in rural areas. Same standards of blood collection were applied for all the scenarios.

After collection, these fresh venous blood samples were transported, at 4℃ temperature, to either local CDC laboratories or township level hospitals near the study sites. One tube of a 4 mL whole blood was collected to obtain plasma (yield a little under 2 mL of plasma) and buﬀy coat. The plasma was then stored in three 0.5 mL cryovials and the buﬀy coat in a separate cryovial. These cryovials were then immediately stored frozen at -20℃ and transported to the Chinese CDC in Beijing within 2 weeks where they were placed in a deep freezer and stored at -80℃ until assay at Youanmen Center for Clinical Laboratory of Capital Medical University. CMU laboratory has regular external quality assessment organized by the Chinese Ministry of Health and conducts assay quality control samples on a daily basis. This laboratory has excellent performance during annual evaluation by External Quality Assurance (EQA) Program organized by the National Center for Clinical Laboratories, China Ministry of Health.

The assay methods for the kidney function measurements used in this laboratory, coeﬃcients of variation (CVs), and detection limits are summarized in Table S1.

**S1.2 Equations for the estimation of glomerular filtration rates (GFR)**

We applied the two equations used in the previous study to calculate eGFR by SCR (GFRscr) or CYS (GFRcys), based on age and sex (*1*). The two equations have taken considerations of more parameters, and were proved to outperform other eGFR approaches for Chinese adults, based on a comparative study (*2*). The detailed equations are shown in Table S2.

**S1.3 Environmental exposure assessment**

Long-term exposure to PM_2.5_ for the studied population was estimated using hindcast estimator, a machine learning based approach, which integrates the data of historical emissions and satellite remote sensing measurements, and produces the monthly PM_2.5_ concentration across a regular grid of 0.1° × 0.1° (about 10 km × 10 km) over China from 2000 to 2016. Details of this method have been elaborated in a previous study (*3*)^.^ The result of cross validation compared to in-situ observations from the Chinese air pollution monitoring network showed high correlation on monthly (R^2^ = 0.71) and annual resolution (R^2^ = 0.77). To control for any potential confounding effect of climate on kidney function, we also included an estimation of temperature based on the method from a previous study (*4*).

The estimation of environmental variables was then linked with the CHALES database by geocode at city level, as this is the finest scale of residential location in the CHARLS database for public use due to the confidentiality considerations. To generate reasonable estimations of exposure at the city level, we aggregated the gridded maps of PM_2.5_ and temperature according to the administrative boundaries of each city. Annual averages were further calculated based on a 12-month period dating back from the month each subject participated in the survey. The exposure time-window was determined before actual data analysis, referring to previous studies (*5*). We didn’t try any other time-window of exposure to avoid the problem of artificially selecting the significant associations.

**S1.4 Induction of difference-in-difference study**

The approach is designed to evaluate the effect of a policy based on a quasi-experiment and has been previously utilized in studies on the health effects of air pollutants.(*6, 7*) Different from the cross-sectional analysis, e.g. ecological study or case-control study which examine the relationship between health and exposure, the difference-in-difference study infer an association based on the coherence between the health change and exposure change within the same individual. In this respect, the difference-in-difference study design has two major advantages. First, some individual-level factors that were constant or slowly varied with time (e.g. genetic defects), can influence the health outcome but not its temporal variation. Therefore, the design can control for such confounders whether they were measured or not. Second, in a quasi-experiment, we assume the exposure change is driven by anthropogenic interventions, which are less relevant with environmental drivers. For instance, the PM_2.5_ exposure was usually correlated with meteorological variables, but its reduction during the China’s clean air actions was proved to be mostly determined by different measures of emission control.(*8*) In this way, the difference-in-difference study design can avoid the confounding effects from the factors that are usually correlated with an exposure but irrelevant with the intervention of its temporal variations.

**S1.5 Sensitivity tests on the nonlinear and baseline-varying effects of PM_2.5_**

We tested the linearity of the effect, which was presumed as true in above analyses. To test that, we replaced the linear term of ΔPM_2.5_ by a set of penalized spline functions (*f*), shown in Equation below.

ΔBiomarker*_i_* = *f*(ΔPM_2.5,_ *_i_* ) + Δ***x****_i_* ***γ***_1_ + ***x****_i_* ***γ***_2_ + *ε*

Figure S4 showed the results of the nonlinear relationship between PM_2.5_ and each biomarker. Basically, the overall effects estimated by the nonlinear models were consistent with the linear results (Figure S3). According to the pointwise CIs, PM_2.5_ had a significant effect on GFR_scr_, BUN and UA, but not on GFR_cys_. The estimated curvatures indicated that the effect was linear for GFR_scr_, sublinear for BUN and superlinear for UA. The sublinear association suggested that the BUN was more sensitive to the PM_2.5_ reduction (ΔPM_2.5_ < 0) than to the PM_2.5_ increment (ΔPM_2.5_ > 0). In contrast, due to the superlinear curvature of UA, the marginal effect of per-unit PM_2.5_ increment was larger than that of per-unit reduction.

Since the difference-in-difference analysis estimated the associations based on the changes in outcome and exposure, we can further explore how the association was varied by a time-invariant variable, such as the baseline kidney function (e.g., the kidney function determined by genetic factors). Let’s Biomarker*_i_* denote the subject-specific baseline kidney function, which thus is assumed as stable over time. In our main model, the baseline variable (Biomarker*_i_*) is controlled for by the difference-in-difference design, itself. To explore the baseline-varying effect of PM_2.5_ on kidney function, we introduced an interaction between the spline expansion of Biomarker*_i_* and PM_2.5_ into the main model: ΔBiomarker*_i_* = *f*(Biomarker*_i_*_,_) • ΔPM_2.5_,*_i_* + Δ***x****_i_* ***γ***_1_ + ***x****_i_* ***γ***_2_ + *ε*. To get a stable estimation for the baseline kidney function, we utilized the average of two biomarker measurements as the Biomarker*_i_* for each subject.

**S1.6 Sensitivity test on the measurement error**

In the publicly available version of CHARLS datasets, there was no specific address for all the surveyed subjects, in order to protect confidentiality. Therefore, our association models were depended on city-level PM_2.5_ exposures, which thus caused exposure measurement errors. To correct the errors, we applied a well-established bootstrap method(*9*) to take the exposure-generation mechanism into the empirical distribution for the association estimator (*β*). The method can be specified as follows:

1. Estimate the association (*β*) between PM_2.5_ and a biomarker using the fully-adjusted regression (Equation 1);
2. Randomly select a location based on the finest-available exposure data (*i.e.*, a 0.1° × 0.1° pixel of the PM_2.5_ map) within the corresponding city as the address for each subject;
3. Assign *pseudo*-*true* exposure values (PM_2.5_^*^) based on the addresses and the gridded monthly maps of PM_2.5_;
4. Simulate *pseudo*-*true* outcomes (*y*^*^) based on Equation 1 and the *pseudo*-*true* exposures (PM_2.5_^*^);
5. Re-estimate the association (*β*^*^) between *pseudo*-*true* outcomes (*y*^*^) and city-level exposures (PM_2.5_);
6. Repeat the steps 2-5 iteratively to mimic the data-generation procedure, which causes the exposure measurement errors, and utilize the sampled distribution of *β*^*^ as the estimation with error correction.

We conducted a bootstrap-based estimation with 3000 simulations for each biomarker, and calculated median and 95% percentile intervals of the bootstrapped results (*β*^*^).

**S1.7 Urban/rural-area-specific concentrations of PM_2.5_**

Besides residential city, CHARLS also reported the type of residential area (urban or rural area) for each surveyed community. This information can be utilized to lower the error in exposure assessment. Therefore, we derived a product of city-level PM_2.5_ concentrations specifically for urban or rural areas. First, we labelled each 0.1° × 0.1° pixel in the maps of gridded PM_2.5_ concentrations as urban or rural pixel, according to a satellite product indicator for urbanization in China during 1978-2017 (http://data.ess.tsinghua.edu.cn/urbanRuralChina.html). The urbanization product has a fine spatial resolution of 30 × 30 meter, and was aggregated into the 0.1° × 0.1° grid by calculating the corresponding mode numbers. Second, for each city-level geographic unit, we calculated the averages of PM_2.5_ for urban or rural pixels separately. Therefore, for each city-level unit, we had two exposure indicators (i.e., city-level average of urban PM_2.5_, and city-level average of rural PM_2.5_), and assigned them to CHARLS subjects according to combination of two variables: residential city and type of residential community. Using the new exposure data, we re-estimated the linear and nonlinear associations between PM_2.5_ and the four biomarkers.

**S1.8 Biological evidences on association between PM_2.5_ and kidney function**

How PM_2.5_ can cause kidney damage remains unclear due to the paucity of studies on the mechanisms, but it is widely acknowledged that the adverse health effect of PM_2.5_ is not simply limited to the respiratory and circulatory systems but can lead to multiple outcomes related to metabolic disorders and distant organs.(*10*) Several biological pathways are proposed to play important roles, including systemic inflammation, oxidative stress and vascular endothelial dysfunction.(*11*) Kidney, as a highly vascularized organ with critical function of filtering toxic waste from metabolism, might be susceptible to the impact of chemicals introduced to the body by PM_2.5_. A controlled exposure experiment in human clearly demonstrated that ultrafine particles (with diameters <100 nm) can directly pass respiratory-blood barrier and quickly distribute in the whole body with subsequent accumulation in the bladder.(*12*) Besides, PM_2.5_ contains certain chemicals, especially some heavy metal elements, such as cadmium, and lead, have been reported to be detrimental to the kidney.(*13*) Recently, an *in vitro* experiment suggested that the exposure to traffic related particles can lead to the reduction in cell viability in human kidney tubular epithelial cells through increased mitochondrial reactive oxygen species and decreased the mitochondrial membrane potential.(*14*) An *In vivo* study in rats also reported that exposure to diesel exhaust particles may cause kidney oxidative stress, DNA damage, and a reduction in kidney blood ﬂow.(*15*) In addition to the direct nephrotoxicity of particles suggested from experimental evidence, several important risk factors of CKD, such as diabetes, hypertension, CVD, obesity, prematurity and low-birthweight, were all reported to be associated with the exposure to PM_2.5_ which may share mutual mechanisms and aggravate kidney damage indirectly.(*16*)(*13*)

In this study we also included BUN and UA, which few studies have reported before. These two molecular biomarkers in serum are widely used for the clinical evaluation of kidney function.(*17, 18*) BUN reflects the amount of urea, a waste product generated from protein, and UA is the final oxidation product of purine metabolism. The significant PM_2.5_-associated increase in these two biomarkers together with the declined GFR_src_ further strengthened the evidence of kidney dysfunction. To be noted, the changes in biomarkers based on urinary protein was examined in three studies regarding the effect of PM_2.5_, but the results were insignificant.(*19-21*) It was suggested these biomarkers might not be ideal for independent observations of air-pollution related nephrotoxicity, because they are more related to structural abnormalities rather than the functional abnormalities in the GFR.(*19*) More studies are warranted to confirm the PM_2.5_-associated effect on BUN and UA.

**Supplemental Tables**

**Table S1: The assay methods for the kidney function measurements**

| **Biomarker** | **Method** | **Coefficient of variation** | | **Detection Limits** |
| --- | --- | --- | --- | --- |
|  |  | Within-assay | Between-assay |  |
| Creatinine (SCR) | Rate-blanked and compensated  Jaﬀe creatinine method | <1.6% | <2.1% | 0.1 - 25  mg/dL |
| Cystatin C (CYS) | Particle-enhanced turbimetric  assay | < 5% | < 5% | 0.5 - 8  mg/L |
| Blood urea nitrogen  (BUN) | Enzymatic UV method with  urease | <4.4% | <4.1% | 5- 100  mg/dL |
| Uric acid (UA) | UA Plus method | 1.10% | 1.90% | Up to 20  mg/dL |

**Table S2. Equations for GFR estimation**

| **Name** | **Gender** | **SCR** | **CYS** | **Equation** |
| --- | --- | --- | --- | --- |
| GFRscr | female | ≤0.7 |  | 144×(scr/0.7)^0.156^×0.993^age^ |
|  |  | >0.7 |  | 144×(scr/0.7)^-1.057^×0.993^age^ |
|  | male | ≤0.9 |  | 141×(scr/0.9)^0.074^×0.993^age^ |
|  |  | >0.9 |  | 141×(scr/0.9)^-1.057^×0.993^age^ |
| GFRcys |  |  |  | 78.64×cys^-0.964^ |

**Supplemental Figures**

******

**Figure S1 Two waves of health surveys before and after intervention (black bars) with the time-series of monthly ambient PM_2.5_ averages (red lines) across the study period.** X axis refers to the date from January 2009 to December 2016. Y axis on the left refers to the monthly concentration of ambient PM_2.5_. Y axis on the right refers to the sample size of monthly person-visit during each wave of survey.


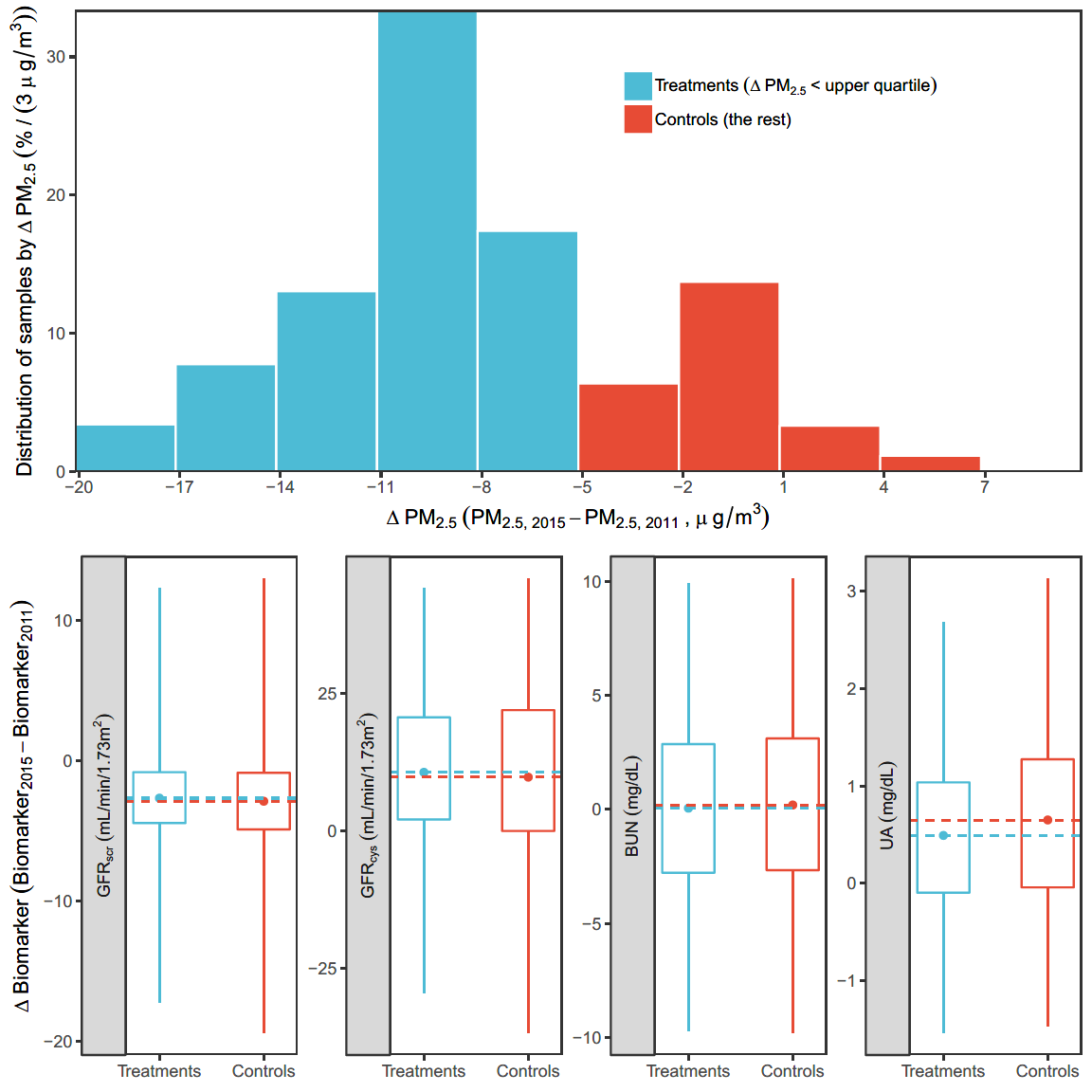


**Figure S2 Illustration of the study design: a preliminary difference-in-difference analysis by treatment and control groups.** Upper panel refers to a histogram of participant sample proportions by the PM_2.5_ reduction they experienced before and after clean air action; ΔPM_2.5_, equals the concentration of PM_2.5_ in 2015 minus the concentration in 2013. Subjects were divided into two groups, namely those who lived in the areas with a ΔPM_2.5_ below its upper quartile (ΔPM_2.5_ < -5.12 μg/m^3^) as the treatment group (blue), referring to a more efficient effect of the clean air actions, and the rest as the control group (red). Lower panel refers to the corresponding changes in the four measured biomarkers, categorized by treatment and control groups.

(a)

(b)

(c)

**Figure S3 Estimated effects of PM_2.5_ on the biomarkers of kidney function by different model runs.** (a) shows the associations estimated from city-level concentrations of PM_2.5_; (b) shows the associations estimated from urban-or-rural-specific city-level concentrations of PM_2.5_. (c) shows the comparison between the two exposure indicators, which are highly correlated with each other (R^2^ = 0.795; 95% CI: 0.789 ~ 0.801). Longitudinal covariates denoted temporal changes in the inconstant variables (i.e., body weight, marriage, drinking, smoking, cooking energy type and indoor temperature maintenance the baseline covariates denoted values of the longitudinal variables in the baseline wave and the constant variables (i.e., residence, sex, education, age at 2011 and average BMI). The fully adjusted model incorporated both the baseline covariates and the longitudinal covariates. The dots denote significant associations, and the circles denote non-significant ones.

(a)

******

(b)

**Figure S4 Estimated nonlinear effects of PM_2.5_ on the biomarkers of kidney function.** (a) shows the nonlinear associations estimated from city-level concentrations of PM_2.5_; (b) shows the nonlinear associations estimated from urban-or-rural-specific city-level concentrations of PM_2.5_. The solid lines denote the pointwise estimates and the dashed lines denote the corresponding confidence intervals.

******

**Figure S5 Estimated baseline-varying effects of PM_2.5_ on the biomarkers of kidney function.** The solid lines denote the pointwise estimates and the dashed lines denote the corresponding confidence intervals. The red dots present population-level averages for baselines (i.e., mean of the two measurements for each individual) of the biomarkers; and the boxplots present distributions of the baselines.

**Figure S6 Estimated effects of PM_2.5_ on the normalized biomarkers of kidney function by different model runs.** Different to the results presented in Figure S3a, the biomarkers are first normalized for each wave, and then their differences are associated to the changes in PM_2.5_ by different models. The sensitivity analysis dealt with the following potential issues: (1) a biomarker doesn’t restrictively follow a normal distribution, and (2) there are potential inconsistency between two waves (For instance, there may be a systematic bias between measurements on the same biomarker in the two waves).

**Figure S7 Estimated effects of PM_2.5_ on the normalized biomarkers of kidney function by different time-windows (i.e., periods of 1-4 years before biomarker measurement) for exposure.**

**References**

1. X. Pei *et al.*, Using mathematical algorithms to modify glomerular filtration rate estimation equations. *PLoS One* **8**, e57852 (2013).

2. L. A. Inker *et al.*, Estimating glomerular filtration rate from serum creatinine and cystatin C. *N Engl J Med* **367**, 20-29 (2012).

3. T. Xue *et al.*, Spatiotemporal continuous estimates of PM2. 5 concentrations in China, 2000–2016: A machine learning method with inputs from satellites, chemical transport model, and ground observations. *Environment international* **123**, 345-357 (2019).

4. T. Xue, T. Zhu, Y. Zheng, Q. Zhang, Declines in mental health associated with air pollution and temperature variability in China. *Nature communications* **10**, 1-8 (2019).

5. J. Bragg-Gresham *et al.*, County-level air quality and the prevalence of diagnosed chronic kidney disease in the US Medicare population. *PloS one* **13**, e0200612 (2018).

6. T. Xue *et al.*, Clean air actions in China, PM2. 5 exposure, and household medical expenditures: A quasi-experimental study. *PLoS medicine* **18**, e1003480 (2021).

7. J. Li *et al.*, Association of long-term exposure to PM2. 5 with blood lipids in the Chinese population: Findings from a longitudinal quasi-experiment. *Environment International* **151**, 106454 (2021).

8. Q. Zhang *et al.*, Drivers of improved PM2.5 air quality in China from 2013 to 2017. *Proc Natl Acad Sci U S A* **116**, 24463-24469 (2019).

9. A. A. Szpiro, L. Sheppard, T. Lumley, Efficient measurement error correction with spatially misaligned data. *Biostatistics* **12**, 610-623 (2011).

10. R. D. Brook *et al.*, Particulate matter air pollution and cardiovascular disease: an update to the scientific statement from the American Heart Association. *Circulation* **121**, 2331-2378 (2010).

11. E.-A. J. I. n. j. Kim, Particulate matter (fine particle) and urologic diseases. *International neurourology journal* **21**, 155 (2017).

12. A. Nemmar *et al.*, Passage of inhaled particles into the blood circulation in humans. *Circulation* **105**, 411-414 (2002).

13. B. Afsar *et al.*, Air pollution and kidney disease: review of current evidence. *Clin Kidney J* **12**, 19-32 (2019).

14. Y. H. Hsu *et al.*, Traffic-related particulate matter exposure induces nephrotoxicity in vitro and in vivo. *Free Radic Biol Med* **135**, 235-244 (2019).

15. A. Nemmar *et al.*, Prolonged Pulmonary Exposure to Diesel Exhaust Particles Exacerbates Renal Oxidative Stress, Inflammation and DNA Damage in Mice with Adenine-Induced Chronic Renal Failure. *Cell Physiol Biochem* **38**, 1703-1713 (2016).

16. P. Romagnani *et al.*, Chronic kidney disease. *Nature reviews Disease primers* **3**, 1-24 (2017).

17. C. Giordano, O. Karasik, K. King-Morris, A. Asmar, Uric acid as a marker of kidney disease: review of the current literature. *Disease markers* **2015**, (2015).

18. S. Lopez-Giacoman, M. Madero, Biomarkers in chronic kidney disease, from kidney function to kidney damage. *World J Nephrol* **4**, 57-73 (2015).

19. T.-C. Chan *et al.*, Long-term exposure to ambient fine particulate matter and chronic kidney disease: a cohort study. *Environmental health perspectives* **126**, 107002 (2018).

20. M. S. O'Neill *et al.*, Airborne particulate matter exposure and urinary albumin excretion: the Multi-Ethnic Study of Atherosclerosis. *Occup Environ Med* **65**, 534-540 (2008).

21. S. Kim, J. Y. Uhm, Individual and Environmental Factors Associated with Proteinuria in Korean Children: A Multilevel Analysis. *Int J Environ Res Public Health* **16**, (2019).
